# Data First: Criminal Courts Linked Data research report.

**DOI:** 10.23889/ijpds.v7i3.1920

**Published:** 2022-08-25

**Authors:** Georgina Eaton, Kylie Hill, Amy Summerfield

**Affiliations:** 1 Ministry of Justice

## Objectives

The Ministry of Justice’s pioneering data linking programme Data First, funded by Administrative Data Research UK, links administrative datasets across the justice system and with other government departments to enable research providing critical new insights on justice system users, their pathways, and outcomes across a range of public services.

## Approach

The first two datasets shared under the Data First project are magistrates’ courts and Crown Court data which have been deidentified, deduplicated and linked to provide a joined-up picture of criminal court defendant and case journeys. Accredited researchers can access this data using the ONS Secure Research Service to conduct research. Administrative Data Research UK has funded four Research Fellows to conduct analysis using this linked data. Additionally, analysts within the Ministry of Justice Data First team have published a research report showcasing the potential of the linked criminal courts data. The presentation will primarily focus on this work.

## Results

The Data First criminal courts datasets have enabled, for the first time, the extent and nature of repeat users to be explored at scale for research. In March 2022, the Ministry of Justice published exploratory analysis of returning defendants and the potential of linked criminal courts data. The key findings of this report will be covered in the presentation. The research demonstrates more than half of defendants returned to the courts within the data period, but this was highest for specific offence groups, including theft, robbery and drug offences. Locality-based analysis on Crown Court defendants highlights important insights on the backgrounds of justice system users, showing an over-representation of defendants residing in the most deprived areas in England and Wales compared to the general population.

## Conclusion

The presentation will demonstrate how linked administrative data available through the ground-breaking Data First programme can be effectively used for research. This insight improves our understanding of individuals in the justice system as well as providing a rich resource to develop the evidence base for government policy and practice.

